# Tumors of the Cerebrum

**Published:** 1907-08

**Authors:** 


**Tumors of the Cerebrum.** Their Focal Diagnosis and Surgical Treatment. By **Charles K. Mills, Charles H. Frazier, William G. Spiller, George E. deSchweinitz,** and **Theodore H. Weisenburg.** Small 8 vo., pp. 35. Philadelphia: Edward Bennock, 3609 Woodland Avenue. 1906.

An extended review of this book is wholly unnecessary because, being a model of its type, it speaks for itself, and no one who has the most casual interest in tumors of the cerebrum, their focal diagnosis and treatment, can afford to pass it by.

It is not a textbook but a collection of seven papers recently written and read before scientific bodies by men who have not their peers anywhere in the fields which they have covered in their work.

An idea of the scope of the papers may be gathered from their titles: The focal diagnosis of operable tumors of the cerebrum, Charles K. Mills, professor of neurology, University of Pennsylvania; Remarks upon the surgical aspects of operable tumors of the cerebrum, Charles H. Frazier, professor of clinical surgery, University of Pennsylvania; Cerebral decompression, with case records and photographs, William G. Spiller and Charles G. Frazier; The ocular symptoms of tumor of the cerebrum, George E. de Schweinitz; Conjugate deviation of the eyes and disorders of the associate ocular movements, Theodore H. Weisenburg; The significance of Jacksonian epilepsy in focal diagnosis with some discussion of the site and nature of the lesions and disorders causing this form of spasm, Charles K. Mills; The motor area of the human cerebrum, its position and subdivision, with some discussion of the surgery of this area, Charles K. Mills and Charles H. Frazier.

The names of these writers is sufficient guaranty of the excellence of the papers and their scientific thoroughness. The bibliography is complete and there is no lack of detail in the description of instruments and technic. The illustrations are of exceptional excellence.

Graceful indeed is the dedication of the book: "To William Williams Keen, M.D., LL.D., and Sir Victor Horsley, F.R.S., and F.R.C.S., in recognition of their great work in cerebral localisation and cerebral surgery and as a mark of personal regard and esteem this little book is dedicated by the authors."

